# Haptoglobin Gene Polymorphism among Sickle Cell Patients in West Cameroon: Hematological and Clinical Implications

**DOI:** 10.1155/2021/6939413

**Published:** 2021-10-20

**Authors:** Christian Bernard Kengne Fotsing, Constant Anatole Pieme, Prosper Cabral Biapa Nya, Jean Paul Chedjou, Samuel Ashusong, Gisele Njindam, Jocelyn Tony Nengom, Georges Teto, Carine Nguemeni, Wilfred Fon Mbacham, Donatien Gatsing

**Affiliations:** ^1^Research Unit of Microbiology and Antimicrobial Substances, Department of Biochemistry, Faculty of Science, University of Dschang, P O. Box 67, Dschang, Cameroon; ^2^Research Unit of Biochemistry of Medicinal Plants, Food Sciences and Nutrition, Department of Biochemistry, Faculty of Science, University of Dschang, P O. Box 67, Dschang, Cameroon; ^3^Laboratory of Biochemistry, Department of Biochemistry and Physiological Sciences, Faculty of Medicine and Biomedical Sciences, University of Yaounde I, P O. Box, Yaounde 1364, Cameroon; ^4^Laboratory for Public Health Research Biotechnology, Department of Biochemistry, University of Yaounde I, Yaounde, Cameroon; ^5^Bafoussam Regional Hospital, P O. Box 980, Bafoussam, Cameroon; ^6^Centre International de Recherche Chantal Biya, Yaounde, Cameroon; ^7^Department of Neurology, University Hospital of Würzburg, Würzburg, Germany

## Abstract

Haptoglobin is a protein involved in protecting the body from the harmful effects of free hemoglobin. The haptoglobin gene exhibits a polymorphism, and the different genotypes do not have the same capacity to combat the free hemoglobin effects. The present study aimed at determining the polymorphic distribution of haptoglobin in sickle cell patients (SCPs) from West Cameroon and their impact on the hematological parameters, as well as clinical manifestations of the disease severity. Haptoglobin genotype of 102 SCPs (SS) and 115 healthy individuals (60 AA and 55 AS) was determined by allele-specific polymerase chain reaction, and the complete blood count was determined using the AutoAnalyser. Results showed that the genotype Hp2-2 was significantly (*p* < 0.05) represented in SS patients (54%) than in controls AA and AS (27% and 29%, respectively), while Hp2-1 was mostly found (*p* < 0.05) in AS (42%) and AA (38%), against 15% in SS. The allelic distribution in SS patients was Hp^2^: 0.613, Hp^1S^: 0.304, and Hp^1F^: 0.084. In AA and AS controls, the proportions of the Hp^1^ and Hp^2^ alleles were similar (around 0.5 each), with 0.282 for Hp^1S^ and 0.218 for Hp^1F^ in AS and 0.283 for Hp^1S^ and 0.258 for Hp^1F^ in AA. The distribution of the haptoglobin genotypes did not reveal any significant difference across hematological parameters and clinical manifestations of disease severity in SCP and controls. SCP with Hp1S-1F genotype presented the highest level of hemoglobin. Although Hp2-2 was more frequent in SS patients, it appeared not to be related to the hematological parameters and to the disease's severity. Further investigations are necessary to explore the impact of Hp polymorphism such as antioxidant, lipid profile, and functionality of some tissues in SCP in Cameroon.

## 1. Introduction

Sickle cell disease (SCD) is the most common genetic disease in the world, affecting more than 50 million people, including 38 million in sub-Saharan Africa [1]. In Cameroon, the prevalence of the sickle cell trait (heterozygous AS) is estimated between 8 and 34% [2], while the prevalence of the disease is between 2 and 3% [3]. The disease kills about 4,000 people each year, all age groups are affected, and young people aged 10 to 29 years represent 89.2% of patients. This makes SCD a real public health problem. SCD is caused by a mutation at the 6th codon of the beta chain of chromosome 11, which results at the protein level in the substitution of glutamic acid by valine [4]. This substitution transforms hemoglobin A (HbA) into hemoglobin S (HbS) which polymerizes under hypoxia. The polymerization of HbS is at the centre of the pathophysiological process of sickle cell anemia (SCA), which is characterized mainly by chronic hemolytic anemia, vaso-occlusive crisis (VOC), and bacterial infections [5]. Chronic hemolysis results in the presence of a large amount of free hemoglobin in the blood, which is linked to increased endothelial adhesion and nitric oxide (NO) depletion [6, 7], leading to a decrease of its vasodilator, antithrombotic, and anti-inflammatory properties [8, 9]. The first line of cleaning free hemoglobin in the blood is a protein called haptoglobin (Hp) [7, 10], which binds directly to free hemoglobin to form a very stable complex haptoglobin-hemoglobin (Hp-Hb), eliminated by macrophages via phagocytosis [6, 10].

Haptoglobin is a *α*2-sialoglycoprotein synthesized by hepatocytes [11]. Its role is to bind the hemoglobin released during hemolytic episodes [12], thus protecting the body from the harmful effects of free hemoglobin. It also has antioxidant, bacteriostatic, NO inhibition, and prostaglandin synthesis properties. Its angiogenic effects are necessary for the proliferation and differentiation of endothelial cells during angiogenesis [13]. This protein is present in all mammals, but its polymorphism has only been reported in humans [6]. Molecular variations in Hp were first suspected by Jayle and Judas in 1946 [14]. In 1955, Smithies [15] identified three phenotypes of haptoglobin, Hp1-1, Hp2-1, and Hp2-2. Smithies and Walker [16] have shown that these phenotypes are controlled by two autosomal alleles Hp^1^ and Hp^2^, located on chromosome 16, locus 22. Later, Connell et al. [17] and Smithies et al. [18] showed that Hp1 can be divided into two subtypes, Hp1S and Hp1F, thus giving rise to a total of 6 different genotypes (Hp1S-1S, Hp1F-1S, Hp1F-1F, Hp2 -1S, Hp2-1F, and Hp2-2) [19].

These different genotypes have different potentials to counter the effects of free hemoglobin in blood [10]. For example, Hp1-1 is biologically more active than others in binding hemoglobin and suppressing its inflammatory effects [13, 20]. Likewise, Melamed-Frank et al. [21] established that patients with Hp2-2 are more prone to oxidative stress than others. Various clinical conditions have been associated with the polymorphism of haptoglobin. Roguin et al. [22] showed that Hp2-2 is associated with myocardial infarction, as a predictor of the severity and extent of infarction damage in patients with different risk factors. Regarding SCD, Ostrowski et al. [23] as well as Moreira and Naoum [24] already reported a strong association between the disease and the Hp1-1 genotype. Other works have suggested that haptoglobin polymorphism may be involved in the pathophysiology of SCA [6, 25]. In 2014, Gueye et al. [26] conducted studies whose results suggest that SS patients with Hp2-2 genotype exhibited significantly lower Hb means compared to Hp1-1 and Hp2-1 subjects. More recently, Chintagari et al. [27] in an *in vitro* study in sickle cell mice showed that Hp attenuates the toxic effects of hem and iron released after hemolysis. Several preclinical studies are underway to determine how haptoglobin can be used as a specific modulator in SCD and to assess its possible side effects.

It is recognized that the prevalence of different Hp genotypes may vary depending on several factors including geographic area and ethnicity [28–30]. A good knowledge of Hp and its polymorphic distribution could help in the fight against SCD. However, to our best knowledge, there are no data on the polymorphism of haptoglobin in sickle cell patients (SCP) in Cameroon. The objective of the present study was, therefore, to determine the polymorphic distribution of haptoglobin genes in SCP from West Cameroon and to evaluate their impact on some hematological parameters in SCP.

## 2. Materials and Methods

### 2.1. Study Design and Subjects

This was a cross-sectional descriptive study conducted at the West Regional Hospital located in Bafoussam (RHB), Cameroon, from September 2018 to November 2019. One hundred and two (102) SS patients (aged 1 to 40 years) regularly in consultation in RHB were recruited for this study. Patients with other pathologies, pregnant women, patients receiving investigational drugs, patients under blood transfusion, and patients with the past three months' history of crisis were not included in the study. In addition, fifty-five (55) subjects with sickle cell trait (AS) and sixty (60) healthy individuals (AA) as controls were included. They were selected in the west region like SS patients, and they were not genetically related to the patients. SS, AS, and AA phenotypes were determined by agarose gel electrophoresis using the kit CELLOGEL. All the participants were informed of the objectives of the study and were requested written informed consent (of their parents or legal guardians) according to the Declaration of Helsinki. The study was approved by the Ethics Review and Consultancy Committee (ERCC) of CAMBIN (Cameroon Bioethics Initiative) with the reference no: CBI/424/ERCC/CAMBIN.

### 2.2. Methods

#### 2.2.1. Sample Collection and Hematological Parameter Measurement

Five millilitres of blood was collected in EDTA tubes and spotted on filter paper for molecular analyses. Complete blood count was carried out immediately after their collection, using the Mindray BC-2800 AutoAnalyser, and semiquantitative immunometric assay of C-reactive protein (CRP) was evaluated by latex agglutination using the DIALAB kit.

#### 2.2.2. DNA Extraction and Haptoglobin Genotyping

DNA was extracted from dried blood spots heated at 100°C in Chelex-100 in buffered Tris-EDTA as previously described by Plowe et al. [31]. Blood spots on the filter paper were excised with a sterile pair of surgical scissors. The DNA was stored in Tris-EDTA buffer at −20°C until Hp genotyping was performed. Hp genotyping was performed by allele-specific PCR as previously described by Yano et al. [19]. PCR was performed in a 25 *µ*L reaction mixture consisting of 10 mM Tris-HCl (pH 9.0), 50 mM KCl, 0.1% Triton X-100, 2.5 mM MgCl_2_, 200 *µ*M of each dNTP, 1.5 U of Taq DNA polymerase, and 0.2 *µ*M of each primer with 1 *µ*L of DNA extract. Cycling conditions were as follows: 95°C for 3 min, followed by 35 cycles of 94°C for 40 sec, 58°C for 1 min, and 72°C for 2 min. Final extension was carried out at 72°C for 5 min, after which PCR products were stored at 4°C for immediate use or −20°C for long-term use. The T3 thermal cycler (Biometra, UK) was used for the PCR amplification. PCR products were electrophoresed at 50 V for 1 h on 1.2% agarose gels and stained with ethidium bromide, and the Hp genotypes were determined by observing the DNA fragments under UV light.  Table 1 shows the sequences of primers, and Table 2 shows the combinations of the primers and predicted sizes of DNA fragments amplified in each reaction.

## 3. Data Analyses

Data were analysed using Statistical Package for the Social Sciences 20 (SPSS, IBM). The frequencies of the genotypes and individual alleles are presented as percentages. Comparisons were made within and between groups using the chi-square test associated with the *z*-test (Bonferroni adjustment) and one-way ANOVA. According to the size of a population group which was above thirty as well as the descriptive explored test of Levene, statistical tests for parametric tests were used. The significant difference was considered at *p* < 0.05.

## 4. Results

Table 3 shows the distribution of the study population according to age and sex. The sex ratio (M/F) and the mean age of SS patients are 1.04 and 9.5 years, respectively.

The hematological parameters of the different group populations are shown in Table 4. SS patients present significantly low levels of hemoglobin, red blood cells, and hematocrit and significantly high levels of white blood cells, lymphocytes, monocytes, granulocytes, and platelets compared to AS and AA.

Haptoglobin genotype and allele frequency distribution in the study population are shown in Table 5. Out of 217 participants, the most common Hp genotype was Hp2-2 (40%). Among those carrying the Hp^1^ allele, the frequencies were 19.4%, 17.1%, 11.1%, 9.7%, and 2.8%, respectively, for Hp1S-1S, Hp2-1S, Hp2-1F, Hp1F-1F, and 1S-1F, which gave the following allelic distribution: Hp^2^: 0.541, Hp^1S^: 0.293, and Hp^1F^: 0.166. According to the chi^2^ test associated with Bonferroni adjustment, the Hp2-2 genotype was significantly more represented in SS patients (54%, *p* < 0.01) than in controls AA (27%) and AS (29%), while Hp2-1 was mostly found significantly in AS (42%, *p* < 0.001) and AA (38%, *p* < 0.001), respectively, than in SS (15%). Regarding intragroup Hp allele frequency distribution, Hp^2^ was the most present allele in SS with a proportion of 0.61. We found a proportion of Hp^1^ of 0.38 in the same group (with 0.30 for Hp^1S^ and only 0.08 for Hp^1F^), while the proportions of the Hp^1^ and Hp^2^ alleles in AA and AS controls were similar (around 0.5 each) (Table 5).

The analysis of the variations of hematological parameters according to the Hp genotype in SS patients showed no significant difference in SCP (Table 6). Despite the nonsignificance, SCPs with the Hp1S-1F genotype presented the highest level of hemoglobin.

We analysed the variations of clinical manifestations of the severity of SCD (VOC occurrence, number of hospitalizations, sanguine transfusion, severity of anemia, CRP, and body mass index (BMI)) according to the Hp genotype in SS patients (Table 7). We found no significant difference between the different Hp genotypes in relation to these indicators of the severity of the disease in SCP (*p* > 0.05) for all indicators.

## 5. Discussion

The results of this study show that the sex ratio (M/F) is 1.04 in the SS group underlining that SCD distribution is independent of sex. Indeed, SCD is a genetic disease with recessive autosomic transmission; therefore, there is no distinction between men and women. The majority of patients were young (mean age: 9.5 years) with 90.23% of SCP aged below 18 years. This could be explained by the early mortality of SCD patients. Indeed, according to Houwing et al. [32], more than half of SCD patients die before the age of 5 years in sub-Saharan Africa. These results are in agreement with those of Dahmani et al. [33] where the most affected age groups in SCP were children under 10 years of age.

The present study revealed that the most common Hp genotype in the population of West Cameroon is Hp2-2 (40%), followed by Hp1-1 (32%) and Hp2-1 (28%). This distribution is different from that obtained by Constans et al. [34] in a study which investigated protein polymorphisms in Pygmy Bi-Aka, a population of the east region of Cameroon. They obtained a majority of Hp2-1 (47% against 41% for Hp2-2 and 12% for Hp1-1). These results are also different from those obtained by Destro-Bisol et al. [35] which revealed a predominance of Hp1-1 (56%) against 5% and 39% for Hp2-2 and Hp2-1, respectively, in a study focused on serum genetic polymorphism among the Bakakas, a population of the littoral region of Cameroon. These differences could be explained by the ethnic and geographic segregation (west, east, and littoral regions of Cameroon). According to several authors, the polymorphic distribution of Hp varies according to the geographical area [6, 28–30]. Our present results confirm this geographical variability.

Anemia observed in SCP, expressed by low levels of Hb (7.49 g/dL) and RBC (2.98 × 10^12^/L), following the chronic hemolysis present on these patients. This could be explained by the rigidity of the sickle RBC, which consequently has a reduced lifespan average, about 10 to 20 days instead of 120 days like normal RBC. Indeed, in case of deoxygenation, there is polymerization of HbS causing damage to the erythrocytes membrane that becomes rigid and fragile, thus leading to their hemolysis [32]. These results are in agreement with those of Tshilolo et al. [36] in Democratic Republic of Congo (RDC) and Mick et al. [37] in RDC and Dahmani et al. [33] in Morocco, which showed significantly low levels of Hb and RBC in SS patients compared to controls.

The current study is the first to show the Hp genotype distribution among SCP in Cameroon. It revealed that the Hp2-2 genotype is significantly more represented in sickle cell patients (54%), while in AS and AA, Hp2-1 is significantly more represented (42% and 38%, respectively). For the allele frequency distribution, Hp^2^ is the most frequent in SS patients (0.62). These results are similar to those obtained by Adelike and Haider [6] in Kuwaiti patients. Their research showed that the most frequent genotypes were Hp2-2 (52%) in SS patients and Hp2-1 (49%) in AA controls, and the frequency of Hp^2^ allele in SS patients was 0.74. However, these are different from those of Olatunya et al. [38] in Nigeria, Khalid and Khalil [39] in Sudan, Moreira and Naoum [24] in Brazil, and Ostrowski et al. [23] in USA, where the most frequent Hp genotype in SS patients was Hp1-1 (43%, 68%, 36%, and 72%, respectively). Furthermore, the previous work of Bruna et al. [40] in Brazil showed Hp2-1 as the most frequent Hp genotype in SS patients (57%). This multiplicity and the variability of results highlight the need for more studies among many cohorts with diverse ethnic backgrounds to provide more understanding on the Hp polymorphism distribution in SS patients. Since a large body of evidence suggests that the Hp^2^ allele is a major susceptibility gene for (i) the development of vascular complications such as coronary artery and cardiovascular disease [41], (ii) the development of oxidative stress [21], and (iii) for inflammatory status [20], SS patients with the Hp2-2 genotype require enhanced attention and specific healthcare management.

This study also allowed us to examine the association of the Hp polymorphism with frequent vaso-occlusive crisis (VOC), hospitalization, transfusion, and hematological parameters. No significant difference has been found between these parameters according to Hp genotypes. Regarding the frequencies of VOC, these results are similar to those of Adelike and Haider [6] who observed no significant difference in the frequencies of the Hp alleles and no significant association of the Hp^2^ allele with increased VOC frequency in Kuwaiti SS patients. Concerning the hematological profile, the results obtained from the present study corroborate those of Bruna et al. [40], Khalid and Khalil [39], and Olatunya et al. [38] who did not find any significant difference between hematological profiles regarding Hp genotypes. Nevertheless, Gueye et al. [26] in Senegal found that SS patients with the Hp2-2 genotype exhibited significantly lower Hb means compared to Hp1-1 and Hp2-1 subjects. They justified their results by explaining that the Hp2-2 genotype was less effective in binding Hb and neutralizing its oxidative power. Therefore, the patients with Hp2-2 would be more predisposed to severe anemia compared to subjects with other genotypes. Despite the latter, they also indicated that their results should be interpreted with caution, especially by taking into consideration factors such as the HbF level, which could interfere in the modulation of the severity of anemia [26].

Despite the lack of influence of other genotypes on the hematological parameters, SCPs with the Hp1S-1F genotype presented the highest levels of hemoglobin, albeit from only 1 result. Because of the small size of the Hp1S-1F genotype, we cannot make a conclusion about a protective effect of the Hp1S-1F genotype. One limitation of the current study could be the small-size sample. A larger sample size could have more power and could probably yield more associations with certain parameters among SCP.

## 6. Conclusions

Our study describes the Hp genotype distribution among sickle cell patients in Cameroon. It reveals that the Hp2-2 genotype is the most represented one in SS patients in West Cameroon. This work did not reveal any significant association between the haptoglobin gene polymorphism and the hematological parameters, as well as some severity manifestations of the disease; however, SCP with the Hp1S-1F genotype presented the highest level of hemoglobin. These results suggest that the polymorphism of the haptoglobin gene may not be a major genetic contributor of sickle cell anemia severity. More prospective studies need to be conducted to explore Hp polymorphism and several other SCA manifestations in sickle cell patients in Cameroon.

## Figures and Tables

**Table 1:** Nucleotide sequences of the primers used.

Primer	Oligonucleotide sequence (5′ – 3′)
F3	CAGGAGTATACACCTTAAATG
S2	TTATCCACTGCTTCTCATTG
C42	TTACACTGGTAGCGAACCGA
C72	AATTTAAAATTGGCATTTCGCC
C51	GCAATGATGTCACGGATATC

**Table 2:** Primer set for PCR.

Reaction	Primer sets (F-R)	Target alleles	Predicted size (bp)
Reaction 2	F3-C42	Hp^2^	935
Reaction S	C51-S2	Hp^1S^	1200
Reaction F	F3-C72	Hp^1F^	1400

**Table 3:** Age and sex distribution of the study population.

Variables		SS (*n* = 102)	AS (*n* = 55)	AA (*n* = 60)
Gender	Male	52 (51%)	10 (18.2%)	28 (46.7%)
	Female	50 (49%)	45 (81.8%)	32 (43.3%)
Sex ratio (M/F)		1.04	0.22	0.88
Age (years)	Mean ± SD	9.5 ± 7.1	25.1 ± 16	14.6 ± 7.2
	Range	1–40	2–55	1–36

SD: standard deviation.

**Table 4:** Hematological parameters among the study groups.

Group	SS	AS	AA	*p* value
WBC (×10^9^/L)	18.06 ± 8.45^a^	7.55 ± 3.96^b^	6.19 ± 1.81^b^	≤0.001^∗^
Lymph (×10^9^/L)	8.38 ± 4.68^a^	3.50 ± 2.29^b^	2.85 ± 0.94^b^	≤0.001^∗^
Mon (×10^9^/L)	1.89 ± 0.85^a^	0.78 ± 0.52^b^	0.57 ± 0.21^c^	≤0.001^∗^
Gran (×10^9^/L)	7.79 ± 5.23^a^	3.26 ± 1.57^b^	2.77 ± 1.04^b^	≤0.001^∗^
Lymph%	46.2 ± 10.5^a^	45.5 ± 8.6^a^	46.3 ± 8.4^a^	0.880
Mon%	10.9 ± 3.5^a^	10.4 ± 3.2^a,b^	9.3 ± 2.3^b^	≤0.005^∗^
Gran%	42.8 ± 10.8^a^	44.0 ± 8.6^a^	44.4 ± 8.6^a^	0.523
Hb (g/dL)	7.49 ± 1.32^c^	11.66 ± 2.03^b^	12.5 ± 1.10^a^	≤0.001^∗^
RBC (×10^12^/L)	2.98 ± 0.68^c^	4.77 ± 0.84^b^	5.08 ± 0.43^a^	≤0.001^∗^
HCT (%)	27.0 ± 5.0^c^	40.5 ± 6.6^b^	43.0 ± 3.36^a^	≤0.001^∗^
MCV (fL)	91.1 ± 12.8^a^	85.7 ± 8.1^b^	85.0 ± 5.6^b^	≤0.001^∗^
MCH (pg)	25.64 ± 3.05^a^	24.55 ± 2.32^b^	24.46 ± 2.17^b^	≤0.001^∗^
MCHC (g/dL)	27.91 ± 1.50^b^	28.69 ± 0.86^a^	28.99 ± 0.72^a^	≤0.001^∗^
RDW-CV%	23.4 ± 3.4^a^	16.2 ± 3.9^b^	14.3 ± 1.2^c^	≤0.001^∗^
RDW-SD (fL)	76.5 ± 13.4	50.5 ± 11.9	45.7 ± 4.3	≤0.001^∗^
PLT (×10^9^/L)	469 ± 181^a^	333 ± 150^b^	283 ± 78^b^	≤0.001^∗^
MPV (fL)	9.83 ± 0.67^b^	10.14 ± 0.66^a^	10.05 ± 0.84^a,b^	≤0.05^∗^
PDW	14.7 ± 0.3	14.5 ± 0.2	14.6 ± 0.2	0.175
PCT%	7.51 ± 0.9	0.31 ± 0.09	0.28 ± 0.07	0.522

WBC: white blood cells; RDW: red cell distribution width; PDW: platelet distribution width; PCT: platelet crit; a, *b*, and *c*: values with different letters in the same line are significantly different at *p* < 0.05; ANOVA with post hoc Dunnet T3.

**Table 5:** Haptoglobin genotype and allele frequency distribution in the study population.

Groups		SS (*n* = 102)	AS (*n* = 55)	AA (*n* = 60)	*p* value
Hp genotypes	Hp 1-1	32 (31.4%)^a^	16 (29.1%)^a,b^	21 (35%)^a,b^	≤0.001^∗^
	Hp 2-1	15 (14.7%)^b^	23 (41.8%)^a^	23 (38.3%)^a^	
	Hp 2-2	55 (53.9%)^a^	16 (29.1%)^b^	16 (26.7%)^b^	
	Hp1S-1S	25 (24.5%)^a,b^	8 (14.5%)^a^	9 (15%)^a^	≤0.001^∗^
	Hp1F-1F	6 (5.9%)^a,b,c^	6 (10.9%)^a^	9 (15%)^a^	
	Hp1S-1F	1 (1%)^a,b,c^	2 (3.6%)^a^	3 (5%)^a^	
	Hp2-1S	11 (10.8%)^a,c^	13 (23.6%)^a^	13 (21.7%)^a^	
	Hp2-1F	4 (3.9%)^c^	10 (18.2%)^a^	10 (16.7%)^a^	
	Hp2-2	55 (53.9%)^a,b^	16 (29.1%)^a^	16 (26.7%)^a^	

Hp allele frequencies	Hp^1S^	0.304	0.282	0.283	
	Hp^1F^	0.084	0.218	0.258	
	Hp^2^	0.613	0.5	0.458	

*a*, *b*, and *c*: values with different letters in the same line are significantly different at *p* < 0.05; SD: standard deviation; ∗: chi^2^ test with the Bonferroni adjustment and Fischer test for small size groups.

**Table 6:** Variation of hematological profile according to the Hp genotype in SS patients.

Parameters	Hp 1S-1S	Hp 1F-1 F	Hp 1S-1 F	Hp 2-1S	Hp 2-1F	Hp 2-2	*p* value
Number of individuals	25 (24.5%)	6 (5.9%)	1 (1%)	11 (10.8%)	4 (3.9%)	55 (53.9%)	
Hb (g/dL)	7.19 ± 1.03	8.23 ± 2.62	8.90	7.54 ± 1.33	7.62 ± 0.70	7.49 ± 1.30	0.532
WBC (×10^9^/L)	17.12 ± 6.24	16.95 ± 4.47	11.90	20.89 ± 13.28	15.22 ± 4.74	18.33 ± 8.82	0.764
RBC (×10^12^/L)	2.94 ± 0.75	3.42 ± 1.25	3.20	3.02 ± 0.56	2.97 ± 0.48	2.94 ± 0.62	0.737
Lymph (×10^9^/L)	8.42 ± 4.26	8.51 ± 2.91	6.50	7.43 ± 1.74	6.17 ± 1.95	8.76 ± 5.56	0.877
Mon (×10^9^/L)	1.76 ± 0.76	1.66 ± 0.85	1.10	1.93 ± 0.70	1.45 ± 0.47	2.01 ± 0.94	0.570
Gran (×10^9^/L)	6.92 ± 2.71	6.76 ± 1.71	4.30	11.51 ± 12.46	7.60 ± 4.04	7.55 ± 3.79	0.223
Lymph%	48.5 ± 10.4	49.4 ± 8.2	54.6	40.6 ± 11.9	41.9 ± 15.2	46.5 ± 10.0	0.300
Mon%	10.7 ± 3.8	9.6 ± 2.7	9.0	10.4 ± 3.9	9.5 ± 1.5	11.5 ± 3.5	0.659
Gran%	40.8 ± 9.7	41.0 ± 8.6	36.4	49.0 ± 13.3	48.6 ± 16.0	42.0 ± 10.3	0.269
HCT (%)	26.33 ± 5.58	29.43 ± 7.97	30.60	26.81 ± 5.08	27.95 ± 3.05	26.99 ± 4.75	0.798
MCV (fL)	91.25 ± 11.17	88.50 ± 9.53	95.90	89.13 ± 7.20	94.95 ± 8.50	91.29 ± 15.25	0.965
MCHC (g/dL)	28.30 ± 1.72	27.73 ± 3.21	29.0	28.18 ± 1.23	27.30 ± 1.02	27.70 ± 1.22	0.586
PLT (×10^9^/L)	458 ± 175	451 ± 307	551	454 ± 202	608 ± 169	463 ± 166	0.733
MPV (fL)	9.92 ± 0.63	9.91 ± 0.50	9.20	10.03 ± 0.56	9.75 ± 0.50	9.76 ± 0.75	0.720
MCH (pg)	25.85 ± 3.85	24.36 ± 2.79	27.80	25.03 ± 2.21	25.85 ± 2.37	25.77 ± 9.96	0.828
RDW-CV%	23.4 ± 3.2	22.7 ± 5.6	19.0	23.3 ± 2.9	22.2 ± 1.2	23.8 ± 3.5	0.727
RDW-SD (fL)	75.6 ± 14.8	73.1 ± 20.7	66.1	74.4 ± 8.3	74.3 ± 9.2	78.0 ± 13.4	0.837
PDW	14.7 ± 0.4	14.6 ± 0.2	14.1	14.7 ± 0.2	14.6 ± 0.2	14.7 ± 0.4	0.591
PCT%	0.42 ± 0.10	0.37 ± 0.24	0.51	0.39 ± 0.08	0.47 ± 0.19	0.43 ± 0.14	0.809

WBC*:* white blood cells; RDW: red cell distribution width; PDW: platelet distribution width; PCT: platelet crit; ANOVA test.

**Table 7:** Some manifestations of the severity of SCD according to the Hp genotype in SS patients.

Parameters		Hp 1S-1S	Hp 1F-1 F	Hp 1S-1 F	Hp 2-1S	Hp 2-1F	Hp 2-2	*p* value
VOC	0	5 (20.8%)	0 (0.0%)	0 (0.0%)	1 (9.1%)	0 (0.0%)	4 (7.7%)	0.353^ɸ^
	1-2	10 (41.7%)	1 (16.7%)	1 (100%)	3 (27.3%)	1 (25%)	25 (48.1%)	
	3 and above	9 (37.5%)	5 (83.3%)	0 (0.0%)	7 (63.6%)	3 (75%)	23 (44.2%)	

Hospitalization	0	8 (33.3%)	1 (16.7%)	0 (0.0%)	5 (45.5%)	1 (25%)	20 (37.7%)	0.756^ɸ^
	1	10 (41.7%)	2 (33.3%)	1 (100%)	5 (45.5%)	1 (25%)	19 (35.8%)	
	2 and above	6 (25%)	3 (50%)	0 (0.0%)	1 (9.1%)	2 (50%)	14 (26.4%)	

Transfusion	0	12 (50%)	4 (66.7%)	1 (100%)	8 (72.7%)	3 (75%)	29 (54.7%)	0.667^ɸ^
	1 and above	12 (50%)	2 (33.3%)	0 (0.0%)	3 (27.3%)	1 (25%)	24 (45.3%)	

Anemia	yes	23 (95.8%)	4 (66.7%)	1 (100%)	11 (100%)	4 (100%)	53 (96.4%)	0.055^ɸ^
	no	1 (4.2%)	2 (33.3%)	0 (0.0%)	0 (0%)	0 (0%)	2 (3.6%)	

Anemia severity^a^	Severe	11 (47.8%)	3 (75%)	0 (0.0%)	3 (27.3%)	1 (25%)	18 (34%)	
	Moderate	11 (47.8%)	1 (25%)	1 (100%)	8 (72.7%)	3 (75%)	33 (62.3%)	0.803^ɸ^
	Mild	1 (4.3%)	0 (0%)	0 (0.0%)	0 (0.0%)	0 (0.0%)	2 (3.7%)	

Type of anemia^b^	Macrocytic	16 (69.6%)	2 (50%)	1 (100%)	5 (45.5%)	2 (50%)	29 (55.8%)	
	Normocytic	6 (26.1%)	2 (50%)	0 (0.0%)	5 (45.5%)	2 (50%)	19 (36.5%)	0.948^ɸ^
	Microcytic	1 (4.3%)	0 (0%)	0 (0.0%)	1 (9.0%)	0 (0.0%)	4 (7.7%)	

Type of hyperleucocytosis^c^	Moderate	9 (40.9%)	2 (33.3%)	1 (100%)	3 (27.3%)	3 (75%)	20 (40%)	
	Plain	6 (27.3%)	2 (33.3%)	0 (0.0%)	6 (54.5%)	0 (0.0%)	15 (30%)	0.711^ɸ^
	Severe	7 (31.8%)	2 (33.3%)	0 (0.0%)	2 (18.2%)	1 (25%)	15 (30%)	

CRP	Negative	17 (73.9%)	2 (50%)	1 (100%)	7 (63.6%)	1 (25%)	18 (39.1%)	0.071^ɸ^
	Positive	6 (26.1%)	2 (50%)	0 (0.0%)	4 (36.4%)	3 (75%)	28 (60.9%)	
BMI (m/kg^2^)		16.60 ± 2.70	17.13 ± 3.67	14.0	16.99 ± 1.60	14.90 ± 0.57	17.14 ± 2.82	0.550^*α*^

VOC: number of vaso-occlusive crisis (VOC) in one year; hospitalization: number of hospitalizations in one year; transfusion: number of sanguine transfusions in one year; a: severe anemia: Hb < 7 g/dL, moderated anemia: 7 g/dL ≤ Hb < 9 g/dL, mild anemia: 9 g/dL ≤ Hb < 11 g/dL; b: macrocytic anemia: MCV > 90 fL, normocytic anemia: 80 fL ≤ MCV ≤ 90 fL, microcytic anemia: MCV < 80 fL; c: moderate hyperleucocytosis: 10 ×10 9/L < WBC > 15 ×10 9/L, plain hyperleucocytosis: 15×109/L ≤ WBC ≥ 20×109/L, severe hyperleucocytosis: WBC > 20 ×109/L; CRP: C-rective protein; BMI: body mass index; ɸ: chi2 test with the Bonferroni adjustment and Fischer test; *α*: ANOVA test.

## Data Availability

Data can be obtained from the corresponding author.
